# A Change—The Result

**Published:** 1868-03

**Authors:** 


### Editorial.

### A CHANGE—THE RESULT.

At the annual meeting of the Ohio Dental College Association, held the first week of March, 1867, it was, by that body, in conjunction with the Board of Trustees, and the Faculty of the Institution, decided to make a change in the course of study, which should consist in a division of the branches taught; constituting a junior and senior department; the former embracing Anatomy, Physiology, Inorganic Chemistry, Metallurgy, and Mechanical Dentistry; the latter, Histology, Microscopy, Pathology, Organic Chemistry, Therapeutics, and Operative Dentistry.

This plan was presented in the last annual announcement, and at the beginning of the session, the classes were organized accordingly, and with a definite understanding that the arrangement would be strictly carried out, which was accomplished as far as possible.

This being the introduction of the method, it was not practicable to draw the lines as close as they will naturally operate in the future, from the fact that some who were previously entitled to enter the senior class were not fully prepared to do so, and consequently had to devote some time to a review of the studies of the junior class. In the future, however, there will be very little or no occasion for such review, especially by those who have been juniors in this Institution.

The juniors devoted almost their entire attention to the branches of that department, being stimulated thereto by the prospect of a thorough examination at the close of the session, a failure to pass which, would prevent their entering the senior class at the next session. Such examination, however, may, at the option of the student, be deferred till the beginning of the next session.

The junior passing a satisfactory examination at the close of his term, receives a certificate to that effect, which entitles him to enter the senior class without further examination, and he has the gratification of knowing his position in the meantime.

In the practical departments the seniors devote their attention to the Operative Department, except some reviews; and the juniors to the Mechanical.

The advantages arising from such an arrangement were abundantly manifest. First, there is less liability of over-exertion on the part of the student. It need hardly be stated that ten or twelve different branches or studies can not be successfully or profitably engaged in at once by one person, whatever may be his talent, industry and endurance. Four or five branches are quite sufficient for any one. By this method the work is far better accomplished than by the former, as the examinations clearly demonstrate. Again, the student finds much encouragement in the fact that he is making appreciable and definite progress. There is also a stimulus in the fact, that he has a definite object to attain at the close of his session, which is to him an object of as much interest as the close of the session to the senior.

The course indicated above, was in the outstart wholly experimental, and was not taken up without some anxiety; but it has been successful and satisfactory beyond expectation. T.

